# Blockchain-Powered LSTM-Attention Hybrid Model for Device Situation Awareness and On-Chain Anomaly Detection

**DOI:** 10.3390/s25154663

**Published:** 2025-07-28

**Authors:** Qiang Zhang, Caiqing Yue, Xingzhe Dong, Guoyu Du, Dongyu Wang

**Affiliations:** 1 National Energy Group, Shuohuang Railway Development Co., Ltd., Beijing 062356, China; 11072702@ceic.com (Q.Z.); 11110588@ceic.com (C.Y.); 2 School of Artificial Intelligence, Beijing University of Posts and Telecommunications, Beijing 100876, China; dongxingzhe@bupt.edu.cn (X.D.); duguoyu@bupt.edu.cn (G.D.)

**Keywords:** blockchain, LSTM–Attention hybrid model, Industrial IoT, anomaly detection, smart contracts

## Abstract

With the increasing scale of industrial devices and the growing complexity of multi-source heterogeneous sensor data, traditional methods struggle to address challenges in fault detection, data security, and trustworthiness. Ensuring tamper-proof data storage and improving prediction accuracy for imbalanced anomaly detection for potential deployment in the Industrial Internet of Things (IIoT) remain critical issues. This study proposes a blockchain-powered Long Short-Term Memory Network (LSTM)–Attention hybrid model: an LSTM-based Encoder–Attention–Decoder (LEAD) for industrial device anomaly detection. The model utilizes an encoder–attention–decoder architecture for processing multivariate time series data generated by industrial sensors and smart contracts for automated on-chain data verification and tampering alerts. Experiments on real-world datasets demonstrate that the LEAD achieves an $F_{0.1}$ score of 0.96, outperforming baseline models (Recurrent Neural Network (RNN): 0.90; LSTM: 0.94; and Bi-directional LSTM (Bi-LSTM, 0.94)). We simulate the system using a private FISCO-BCOS network with a multi-node setup to demonstrate contract execution, anomaly data upload, and tamper alert triggering. The blockchain system successfully detects unauthorized access and data tampering, offering a scalable solution for device monitoring.

## 1. Introduction

With the increasing scale of industrial equipment and the widespread deployment of sensors, a large volume of heterogeneous time series data (e.g., temperature, vibration, current, pressure) is continuously collected from the Industrial Internet of Things (IIoT). These sensor streams reflect changes in the operational status of devices and, when properly analyzed, can support timely fault detection, improve equipment reliability, and reduce operational costs [1].

To extract actionable insights from such data, the concept of situation awareness—including perception, comprehension, and prediction—provides a structured framework [2]. In the context of industrial devices, this translates into monitoring operational conditions, identifying abnormal behaviors, and predicting future faults based on temporal sensor patterns.

Many mathematical statistics or machine learning methods have emerged to analyze and process industrial equipment data. Models can be trained on normal and abnormal data and then used to detect abnormalities [3]. The operation status of the equipment is often a dynamic process, and the data collected by the sensors have temporal characteristics. Traditional methods based on static data analysis are difficult to capture the evolution trend of equipment status. As a deep learning model that can handle long-term sequence dependencies, Long Short-Term Memory Networks [4] (LSTMs) are widely used in the operation state awareness of industrial equipment. LSTMs can automatically extract features from time series data and identify equipment status trends so as to achieve more accurate fault detection and prediction.

Considering the increasing complexity of industrial device data and the rarity of abnormal instances in real-world scenarios, traditional LSTM models often suffer from overfitting to normal patterns and fail to detect novel or subtle anomalies [5]. Also, it is difficult to rely on machine learning models alone to ensure the security and trustworthiness of data. To prevent data from being tampered with during transmission and storage, blockchain technology can be used in conjunction with an LSTM model. Despite extensive research efforts, two core challenges persist: fault instances are rare and evolve dynamically, making traditional machine learning methods insufficient for high-accuracy anomaly detection; and current systems lack secure, tamper-resistant mechanisms for storing and verifying sensitive device status data.

To address these challenges, we propose a hybrid system that combines deep learning with blockchain technology for device situation awareness. Specifically, we design an LSTM-based encoder–attention–decoder model to capture long-term dependencies in multivariate time series data and accurately detect faults. Meanwhile, we integrate smart contracts on a blockchain to securely record and verify device status and trigger alerts in real-time. According to the data collected by various devices, we can judge their operation status by analyzing their changes in the time series, and then make corresponding decisions. In our model, the running state is mainly divided into two modes: suspicious mode and anomalous mode. The main contributions of our research are described below.

- We propose LEAD, a novel LSTM–Attention-based encoder–decoder model for time series anomaly detection in industrial devices. The model effectively addresses the challenge of detecting rare anomalies and improves the $F_{0.1}$ score to 0.96, outperforming existing RNN, LSTM, and BI-LSTM baselines.
- We design and implement a blockchain-based smart contract mechanism that ensures secure, tamper-resistant, and automated recording of device data and anomaly results. The system is capable of detecting unauthorized uploads, tampering attempts, and generating alerts.

The remainder of the article is organized as follows: Section 2 reviews the work on device state-aware prediction using LSTM-related methods. In Section 3, a method for anomaly detection of time series data based on LSTM combined with an attention mechanism is proposed. In Section 4, we explain how to deploy an auto-detection module on the blockchain to implement on-chain data tampering alarms through smart contracts. In Section 5, we analyze and discuss the results of experiments on real datasets using the RNN, LSTM, BI-LSTM, and LSTM–Attention methods. On-chain anomaly detection and tamper alarms were demonstrated to verify security and effectiveness. Section 6 summarizes the content of this study.

## 2. Related Works

Time series anomaly detection has evolved from traditional statistical methods to machine learning paradigms. Early approaches, such as support vector machines [6], offered simplicity, but struggled with nonlinear temporal dependencies, while basic recurrent networks [7] improved sequence modeling at the cost of gradient vanishing in long-range predictions. The emergence of LSTM-based architectures addressed these limitations, establishing dominance in industrial applications. Bontemps et al. [8] pioneered neural prediction error thresholds for collective anomalies, yet their fixed error thresholds lacked adaptability to dynamic operational environments. Malhotra et al. [5,9] overcame labeled data scarcity through LSTM autoencoders, but their reconstruction-based approach risked overfitting to normal patterns, limiting detection of novel anomaly types. Park, based on the work of Malhotra et al. [9], implemented EncDec-AD (RNN) [10]. Subsequent unsupervised variants like Provotar et al.’s autoencoder [11] and Ergen et al.’s variable-length sequence analysis [12] enhanced generalization, albeit at the expense of interpretability in decision boundaries.

Hybrid architectures emerged to balance robustness and sensitivity. Lin et al. [13] integrated VAEs with LSTMs, leveraging VAE’s local feature generation and LSTM’s temporal coherence for multi-scale detection. However, their disjointed training of VAE and LSTM modules introduced optimization complexity. Park et al. [14] combined autoencoders with LSTM ensembles, achieving superior multivariate fault diagnosis, but requiring prohibitive computational resources for real-time deployment. The attention mechanism in Kong et al.’s AMBi-GAN [15] adaptively weighted bidirectional LSTM features, yet its GAN-based framework suffered from mode collapse in low-diversity industrial datasets. Complementing these, dynamic thresholding techniques addressed false positive trade-offs: Hundman et al. [16] introduced nonparametric thresholds for spacecraft telemetry, adapting to seasonal patterns, but requiring manual sensitivity tuning, while Wang et al.’s BiLSTM–Attention model [17] automated context-aware thresholds through learned data distributions. Wen and Li [18] proposed an LSTM–Attention–LSTM model for time series forecasting, employing dual LSTMs as encoder–decoder with interleaved attention to capture long-range dependencies in non-stationary data.

In this study, we focus on evaluating the effect of integrating attention mechanisms and blockchain with classical RNN and LSTM-based encoder–decoder models, which are representative baselines for time series anomaly detection. While alternative architectures such as GRU and BiLSTM also offer promising capabilities, we reserve these for future work in order to maintain a clear focus on the contributions of attention and secure model integration.

The integration of blockchain technology within the Industrial Internet of Things (IIoT) has garnered significant research interest [19,20,21,22]. Blockchain frameworks, like Li et al.’s data sharing model [23] and Griggs et al.’s medical monitoring system [24], ensure tamper-proof data integrity through smart contracts. However, their consensus mechanisms incurred latency incompatible with time-sensitive anomaly responses. Lightweight diagnostic alternatives emerged: Zhang et al. [25] exploited Raft logs for near-zero-overhead distributed storage diagnosis, but limited detection to consensus-related failures, while ScalaLog [26] circumvented log parsing via LLM-based RAG, enabling scalable failure diagnosis at the cost of dependency on pre-trained language models’ domain adaptability.

Table 1 describes the evaluation criteria that different methods emphasize. In order to address the shortcomings of existing methods, this paper proposes an LSTM Encoder–Attention–Decoder method for time series prediction by inserting a multi-head self-attention module, which improves detection sensitivity and interpretability by highlighting the critical temporal segments that contribute most to anomalies. Compared to complex hybrid architectures like VAEs or GANs, LEAD remains lightweight and more stable in training. At the same time, there are still problems such as insufficient data trustworthiness and lack of automation performance. Due to the requirements of the industrial field, this paper designs a blockchain awareness mechanism to realize the functions of safe storage and anomaly detection through smart contracts. Our work targets device-level anomaly detection and data integrity assurance using blockchain smart contracts. By embedding anomaly scores and detection results directly on-chain, the proposed system extends fault observability to data-origin faults.

## 3. LSTM-Based Encoder–Attention–Decoder (LEAD) Architecture

This section first introduces the basic architecture of the LSTM model, and then specifically explains the LSTM-based Encoder–Attention–Decoder (LEAD) structure. Next, it explains the training and testing modes of this model, as well as the indicators established for equipment time series fault detection for actual anomaly observation.

### 3.1. Proposed Model Architecture

This study proposes an LEAD model for anomaly detection and device situational awareness of time series data from devices. Set time series X={x(1),x(2),…,x(L)}. Each data point $x\left(i\right)\in R^{m}$ represents the observation value of the *m* dimensional sensor reading at time $t_{i}$. Due to the multidimensional and strong temporal correlation characteristics of industrial equipment data, we extract fixed-length subsequences from long time series through sliding windows to construct a dataset for model training and detection.

**Figure 1:**
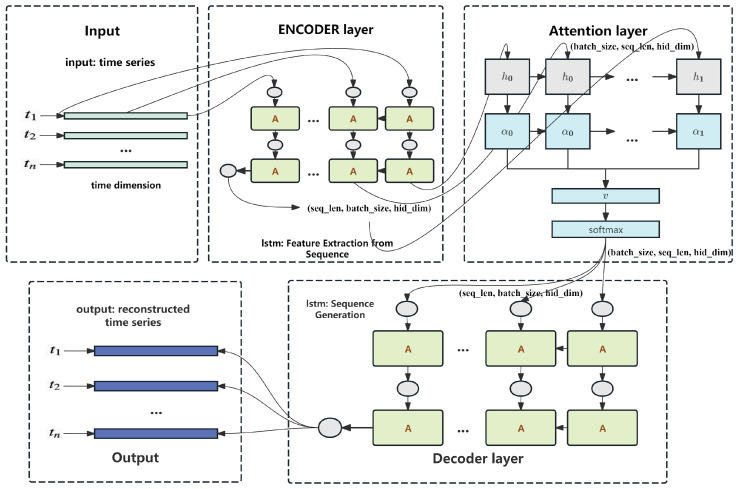
Detailed workflow of the LEAD algorithm.

As illustrated in Figure 1, the LEAD architecture consists of an LSTM-based encoder, a self-attention mechanism, and an LSTM-based decoder. The encoder captures the temporal dependencies of the input sequence and transforms it into a sequence of hidden states. These hidden states are passed through a multi-head self-attention module, which assigns dynamic weights to different time steps, enabling the model to focus on informative moments that may indicate early signs of faults. The attention-weighted representation is then input to the decoder, which reconstructs the original sequence in a step-by-step manner.

During training, the model minimizes the reconstruction error between the input and output sequences, effectively learning the normal temporal patterns of the system. In the inference phase, the reconstruction error for each time step is computed and used to derive an anomaly score a(i) for each data point x(i). A higher anomaly score indicates a greater deviation from the learned normal pattern, suggesting a higher likelihood of fault or abnormal behavior.

### 3.2. Prediction Model Using LEAD

**Figure 2:**
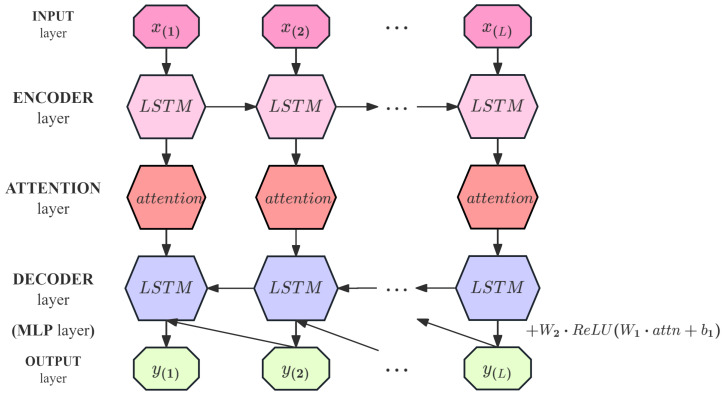
Prediction process for X={x(1),x(2),…,x(L)} of LEAD.

We employ the LEAD to train and predict normal time series. Figure 2 depicts the five main layers of the LEAD. The encoder learns a fixed-length vector representation of the input time series. The LSTM units extract the timing features and generate the hidden state. The attention mechanism assigns different weights at each time step, allowing for the model to focus on various parts of the sequence. The decoder utilizes the encoder’s output representation along with the attention-weighted historical information to predict the time series, relying on the current hidden state and the prediction from the previous time step. This layer is attached with an MLP branch, with the LSTM layer decoding the encoded features and then further extracting the features using MLP. The LSTM decoder output and the MLP branch output are fused outputs.

As Figure 2 shows, given a time series *X* = {x(1),x(2),…,x(L)}, $h_{E}\left(i\right)$ represents the hidden state of the encoder at time $t_{i}$, where $h_{E}\left(i\right)\in R^{c}$ and *c* is the number of LSTM units in the encoder’s hidden layer. The encoder and decoder are jointly trained to reconstruct the target time series in reverse order (similar to Malhotra et al., 2016) [5], i.e., the target time series is {y(L),y(L−1),…,y(1)}. The decoder uses x(i) as input to obtain the state $h_{E}\left(i\right)$. The final hidden state $h_{E}\left(L\right)$ of the encoder is used as the initial state of the attention layer. The output of the attention layer $h_{E}^{\prime }\left(L\right)$ is fed into the decoder through a fully connected layer. The decoder takes the final hidden state of the encoder $h_{E}^{\prime }\left(L\right)$ as the initial input hidden state $h_{D}\left(L\right)$ of the decoder. The decoder employs LSTM units with the same architecture as the encoder and performs step-by-step reverse prediction. At each step *i* (from i=L to i=1), the decoder uses the previously generated output y(i) as the input to the LSTM to update its hidden state $h_{D}\left(i-1\right)$. Then, the current hidden state $h_{D}\left(i-1\right)$ is used to generate the predicted output y(i−1). This process is performed in an autoregressive manner until the entire reversed prediction sequence {y(1),y(2),…,y(L)} is generated.

### 3.3. Illustrative Example of LEAD Workflow

To clarify the working process of the proposed LEAD model, we provide an illustrative example using a representative time series subsequence from the ECG dataset. Suppose we observe a univariate input segment X={0.15,0.21,0.18,0.17,0.91,0.19,0.20}, where the sudden spike at x(5)=0.91 indicates a potential anomaly.

This sequence is first processed by the LSTM-based encoder, which transforms it into hidden states H={h(1),h(2),…,h(7)}. The self-attention mechanism then calculates attention weights to emphasize the most informative time steps. For example, the weights might be α={0.05,0.10,0.15,0.10,0.40,0.10,0.10}, assigning the highest weight to x(5) due to its abnormal value.

Using the weighted context, the decoder reconstructs the sequence, resulting in $\hat{X}=\left\{0.14,0.20,0.19,0.18,0.42,0.18,0.21\right\}$. The anomaly score at each step is calculated as $a\left(i\right)=|x\left(i\right)-\hat{x}\left(i\right)|$, yielding

(1) a={0.01,0.01,0.01,0.01,0.49,0.01,0.01}.

Since a(5)=0.49 significantly exceeds the threshold, x(5) is flagged as an anomaly. This detection result, along with its timestamp and device metadata, is then recorded on the blockchain via smart contracts to ensure secure and verifiable logging.

### 3.4. LSTM Unit

The LSTM architecture comprises three main gates—forget, input, and output—that regulate the flow of information through time [4]. These gates control what information to discard, update, and expose at each time step. Let $X_{t}$ be the input, $h_{t-1}$ the previous hidden state, and $C_{t-1}$ the previous cell state. The LSTM update equations are as follows:

(2) $$\left\{\begin{array}{cc} f_{t} & =\delta (W_{f}\left[h_{t-1},X_{t}\right]+b_{f})\\ i_{t} & =\delta (W_{i}\left[h_{t-1},X_{t}\right]+b_{i})\\ O_{t} & =\delta (W_{o}\left[h_{t-1},X_{t}\right]+b_{o})\\ L_{t} & =\tanh(W_{c}\left[h_{t-1},X_{t}\right]+b_{c})\\ C_{t} & =f_{t}\times C_{t-1}+i_{t}\times L_{t}\\ h_{t} & =O_{t}\times \tanh\left(C_{t}\right) \end{array}\right.$$

where δ denotes the sigmoid function, and tanh is the hyperbolic tangent function. These equations govern how the LSTM updates its internal cell state and produces the hidden state $h_{t}$ at each time step. Equation (2) summarizes the core components of the LSTM unit:

- $f_{t}$: Forget gate—controls which parts of the previous cell state $C_{t-1}$ should be discarded.
- $i_{t}$: Input gate—determines which new information $L_{t}$ should be added to the cell state.
- $L_{t}$: Candidate memory—represents potential new information for the cell state.
- $C_{t}$: Updated cell state—integrates retained memory and new candidate values.
- $O_{t}$: Output gate—controls which parts of the cell state contribute to the hidden state.
- $h_{t}$: Hidden state—serves as both the output at time step *t* and input for the next step.

#### 3.4.1. Encoder Layer

LSTM is part of encoder, which is responsible for converting the input sequence into a fixed-length context vector. This context vector typically contains information from the input series and captures the dependencies of a longer range in the time series. The encoder’s LSTM processes the input sequence and outputs the final hidden state.

The encoder first receives multidimensional time series data with shape $\left(B,T,D_{e}\right)$, where *B* is the batch size (number of sequences processed together), *T* is the sequence length (number of time steps), and $D_{e}$ is the encoder input dimension (number of features at each time step). A linear transformation is applied to project the input into a dimension-reduced feature space $D_{r}$, which is more suitable for the LSTM layer. The transformation is

(3) $$emb=W_{e}\cdot x+b_{e}$$

where emb is the embedded representation, $W_{e}\in R^{D_{e}\times D_{r}}$ is the weight matrix, and  $b_{e}\in R^{D_{r}}$ is the bias. After this operation, the data shape becomes $\left(B,T,D_{r}\right)$.

This embedded input is then passed into an LSTM, along with an initial hidden state. The LSTM captures the temporal dependencies and outputs, which are subsequently passed into the attention layer, a hidden state of shape (L,B,H), and an output feature sequence of shape (T,B,H).

#### 3.4.2. Attention Layer

Before applying self-attention, adjust the LSTM output from (T,B,H) to (B,T,H) to fit the attention computation:

(4) $$O\underset{\mathrm{permute}(1,\;0,\;2)}{\to }R^{B\times T\times H}$$

where $O\in R^{T\times B\times H}$ denotes the LSTM output tensor, *T* is the sequence length, *B* is the batch size, and *H* is the hidden dimension.

Self-attention [27] models dependencies between time steps by projecting the input into three matrices: query (*Q*), key (*K*), and value (*V*). The dot-product of *Q* and *K* generates attention scores, which are weighed *V* to produce context-aware representations.

We employ multi-head self-attention [27] to capture diverse feature relationships. Compared to single-head or additive attention mechanisms, multi-head attention allows for the model to jointly attend to information from different representation subspaces at different time steps. This enables the model to better capture complex temporal dependencies and subtle variations across multivariate time series data. The hidden dimension *H* is divided into *m* heads (*m* denotes the number of attention heads), and each head computes independent attention. The outputs are concatenated to form refined features. The matrix transformations are

(5) $$Q=OW_{Q}\in R^{B\times T\times H},\;K=OW_{K}\in R^{B\times T\times H},\;V=OW_{V}\in R^{B\times T\times H}$$

After splitting,

(6) $$Q_{i}\in R^{B\times m\times T\times d},\;d=\frac{H}{m}$$

where *d* is the dimension per attention head.

The attention weights for the *i*-th head are computed as

(7) $$E_{i}=\frac{Q_{i}K_{i}^{⊤}}{\sqrt{d}}\in R^{B\times m\times T\times T}$$

(8) $$A_{i}=\mathrm{softmax}\left(E_{i}\right)V_{i}\in R^{B\times m\times T\times d}$$

where $E_{i}$ represents the unnormalized attention energy and $A_{i}$ is the attention output of the *i*-th head.

Finally, the multi-head outputs are concatenated and linearly transformed:

(9) $$O^{\prime }=\mathrm{Concat}\left(A_{1},\ldots ,A_{m}\right)W_{O}\in R^{B\times T\times H}$$

To align with decoder inputs, the tensor is transposed to (T,B,H)

(10) $$O^{\prime }\underset{\mathrm{permute}(1,\;0,\;2)}{\to }R^{T\times B\times H}$$

where $O^{\prime }$ denotes the refined self-attention output.

#### 3.4.3. Decoder Layer

The input to the decoder is the output from the attention module. The initial hidden state of the decoder is inherited from the final hidden state of the encoder LSTM. By reusing the encoder’s final state, the decoder is provided with contextual information learned during the encoding phase, enabling it to generate coherent and relevant outputs.

The LSTM receives the refined self-attention output $O^{\prime }$ and hidden state *h* as input, applying recurrent computation across the temporal dimension:

(11) $$D=\mathrm{LSTM}\left(O^{\prime },h\right)\in R^{T\times B\times H}$$

where *D* denotes the decoder output tensor, maintaining shape (T,B,H) for compatibility with downstream layers.

A linear projection maps the hidden representation to the target space:

(12) $$D_{\mathrm{proj}}=DW_{\mathrm{dec}}\in R^{T\times B\times D_{d}}$$

where $W_{\mathrm{dec}}\in R^{H\times D_{d}}$ is the projection matrix and $D_{d}$ aligns with the encoder’s input dimension $D_{\mathrm{enc}}$ for potential residual connections.

The attention output $O^{\prime }\in R^{T\times B\times H}$ is processed by an MLP with ReLU activation:

(13) $$M=W_{2}\cdot \mathrm{ReLU}\left(W_{1}O^{\prime }+b_{1}\right)\in R^{T\times B\times D_{d}}$$

where $W_{1}\in R^{H\times h_{m}}$, $W_{2}\in R^{h_{m}\times D_{d}}$ are weight matrices, $b_{1}$ is the bias vector, and $h_{m}$ denotes the MLP’s hidden dimension.

The outputs are combined via weighted averaging:

(14) $$C=\frac{1}{2}\left(D_{\mathrm{proj}}+M\right)\in R^{T\times B\times D_{d}}$$

When residual connections are enabled,

(15) $$F=C+X\;\mathrm{with}\;D_{\mathrm{enc}}=D_{d}$$

where $X\in R^{T\times B\times D_{\mathrm{enc}}}$ is the original input tensor and the equality $D_{\mathrm{enc}}=D_{d}$ ensures dimensional consistency.

The complete prediction workflow is detailed in Algorithm 1. It illustrates the computation flow from input sequence to output prediction, highlighting key operations and corresponding tensor shape transformations at each stage.
**Algorithm 1** LSTM-based Encoder–Attention–Decoder.**Input:** Input sequence $X\in R^{B\times T\times D_{e}}$**Encoder:**  1:E←LinearEncoder(Flatten(X))            ▹$\left(B\;\times \;T,D_{r}\right)$  2:$E\leftarrow \mathrm{Reshape}{\left(E,\left(B,T,D_{r}\right)\right)}^{⊺}$              ▹$\left(T,B,D_{r}\right)$  3:Initialize $h_{0}\in R^{L\times B\times H}$  4:$\left(O_{e},h_{1}\right)\leftarrow \mathrm{LSTM}\left(E,h_{0}\right)$                ▹(T,B,H)**Attention:**  5:$A\leftarrow O_{e}^{⊺}$                        ▹(B,T,H)  6:$Q,K,V\leftarrow A\cdot W_{q},A\cdot W_{k},A\cdot W_{v}$            ▹$W_{\cdot }\in R^{H\times d}$  7:$\mathrm{Attn}\leftarrow \mathrm{softmax}(\frac{QK^{⊤}}{\sqrt{d}})V$               ▹(B,T,H)  8:$S\leftarrow {\mathrm{Attn}}^{⊺}$                       ▹(T,B,H)**Decoder:**  9:$O_{d}\leftarrow \mathrm{LSTMDecoder}\left(S\right)$                ▹(T,B,H)10:$Y_{r}\leftarrow \mathrm{LinearDecoder}\left(O_{d}\right)$                  ▹$\left(T,B,D_{d}\right)$11:$Y_{m}\leftarrow \mathrm{MLP}\left(\mathrm{Flatten}\left(S\right)\right)$**Fusion:**12:$Y\leftarrow \frac{1}{2}\left(Y_{r}+Y_{m}\right)$13:**if** use_residual **then**14:    $Y\leftarrow Y+X^{⊺}$15:**end if**16:$Y\leftarrow Y^{⊺}$                          ▹$\left(B,T,D_{d}\right)$   **Output:** Prediction $Y\in R^{B\times T\times D_{d}}$

#### 3.4.4. Multi-Objective Joint Training Strategy

Similarly to the process of [10], in our approach, the model is jointly optimized using a three-part loss function.

A. Free-Running Loss

In the free-running mode, the model uses its own predictions as input during the decoding phase. This simulates the inference process and enhances the model’s long-term prediction capabilities. However, since errors accumulate over multiple steps, this mode demands better generalization ability from the model.

The free-running loss evaluates long-term prediction stability:

(16) $$L_{\mathrm{fr}}=\frac{1}{N}\sum_{i=1}^{N}{∥{\hat{Y}}_{i}^{\left(\mathrm{fr}\right)}-Y_{i}∥}_{2}^{2}$$

where *N* denotes the number of samples, ${\hat{Y}}_{i}^{\left(\mathrm{fr}\right)}\in R^{T\times D_{d}}$ refers to the free-running prediction of the *i*-th sample, $Y_{i}\in R^{T\times D_{d}}$ refers to ground truth sequence, and ${∥\cdot ∥}_{2}^{2}$ calculates the squared Euclidean norm.

During free-running, predictions are recursively fed as input:

(17) $$Y^{\left(\mathrm{pred}\right)}\leftarrow f_{\theta }\left(Y^{\left(\mathrm{pred}\right)}\right)$$

where $f_{\theta }$ denotes the model with parameters θ, enforcing multi-step prediction capability.

B. Teacher Forcing Loss

Teacher forcing loss measures short-term prediction accuracy:

(18) $$L_{\mathrm{tf}}=\frac{1}{N}\sum_{i=1}^{N}{∥{\hat{Y}}_{i}^{\left(\mathrm{tf}\right)}-Y_{i}∥}_{2}^{2}$$

where ${\hat{Y}}_{i}^{\left(\mathrm{tf}\right)}\in R^{T\times D_{d}}$ uses ground truth inputs:

(19) $$Y^{\left(\mathrm{in}\right)}\to f_{\theta }\left(Y^{\left(\mathrm{in}\right)}\right)$$

This direct feeding mechanism accelerates convergence by minimizing error accumulation.

C. Hidden State Consistency Loss

Aligning hidden representations between modes,

(20) $$L_{\mathrm{hid}}=\frac{1}{N}\sum_{i=1}^{N}{∥H_{i}^{\left(\mathrm{fr}\right)}-H_{i}^{\left(\mathrm{tf}\right)}∥}_{2}^{2}$$

where $H_{i}^{\left(\mathrm{fr}\right)}\in R^{T\times H}$ represents the hidden states from free-running LSTM and $H_{i}^{\left(\mathrm{tf}\right)}\in R^{T\times H}$ represents the hidden states from teacher-forced LSTM.

The hidden states are generated through

(21) $$\left\{\begin{array}{c} H^{\left(\mathrm{fr}\right)}=\mathrm{LSTM}\left({\hat{Y}}^{\left(\mathrm{fr}\right)}\right)\\ H^{\left(\mathrm{tf}\right)}=\mathrm{LSTM}\left(Y^{\left(\mathrm{in}\right)}\right) \end{array}\right.$$

This consistency constraint enhances model robustness against different inference modes.

The difference between the hidden states is penalized, encouraging the model to maintain consistent internal states across the two modes, thus improving stability during inference.

The final loss function is the sum of the three individual losses:

(22) $$L_{\mathrm{total}}=L_{1}+L_{2}+L_{3}$$

This combined loss function balances long-term and short-term accuracy while ensuring consistency between the hidden states, leading to a more robust and stable model during inference.

### 3.5. Anomaly Detection

Inspired by Malhotra’s method [5], we formalize the anomaly detection procedure into three stages. First, assume prediction errors follow a Gaussian distribution during normal operation, and estimate its parameters (mean and variance) from training data. Second, quantify deviations using Mahalanobis distance, which account for feature-scale variations and reduce to a standardized Euclidean distance under independence assumptions. Then, optimize thresholds by maximizing the $F_{\beta }$ score to balance precision and recall. Finally, classify anomalies using a double-threshold rule derived from the optimized criteria. This pipeline ensures statistically grounded detection while operationalizing thresholds for nuanced state awareness (normal/suspicious/anomalous).

#### 3.5.1. Prediction Error Modeling

Suppose that, under normal operating conditions, the prediction error (i.e., the difference between the actual observed value and the predicted value) is of a Gaussian distribution (normal distribution). Let the time series observation at time *t* be denoted by $x_{t}\in R^{d}$, and the model’s predicted output by ${\hat{x}}_{t}\in R^{d}$. The prediction residual is defined as

(23) $$e_{t}=x_{t}-{\hat{x}}_{t}$$

For each feature dimension i∈{1,2,…,d}, it is assumed that the residuals under normal operating conditions follow a Gaussian distribution $e_{t}\left(i\right)∼N\left(\mu_{i},\sigma_{i}^{2}\right)$. The parameters $\mu_{i}=E\left[e\left(i\right)\right]$ and $\sigma_{i}^{2}=\mathrm{Var}\left(e\left(i\right)\right)$ are estimated from the training data, capturing the mean and variance of the residuals for each feature dimension. This Gaussian assumption allows for the model to detect anomalies by measuring deviations from the expected distribution during inference.

#### 3.5.2. Standardized Anomaly Scores

Mahalanobis distance is generally used to measure the degree to which the current forecast error deviates from the normal distribution. For the multivariate prediction error vector $e_{t}={\left[e_{t}\left(1\right),\ldots ,e_{t}\left(d\right)\right]}^{⊤}$ calculated from Equation (23), the Mahalanobis distance of the error vector is defined as

(24) $$s_{t}={\left(e_{t}-\mu \right)}^{⊤}\Sigma^{-1}\left(e_{t}-\mu \right)$$

where $\mu \in R^{d}$ is the mean vector of the training set errors, and $\Sigma \in R^{d\times d}$ is the covariance matrix of the training set errors. Assuming the features are independent:

(25) Cov(e(i),e(j))=0fori≠j,

The covariance matrix Σ will reduce to a diagonal matrix:

(26) $$\Sigma =\left[\begin{array}{cccc} \sigma_{1}^{2} & 0 & \ldots & 0\\ 0 & \sigma_{2}^{2} & \ldots & 0\\ \vdots & \vdots & \ddots & \vdots \\ 0 & 0 & \ldots & \sigma_{d}^{2} \end{array}\right]$$

Under this assumption, the Mahalanobis distance can be reduced to the standardized Euclidean distance of the individual features:

(27) $$s_{t}=\sum_{i=1}^{d}\frac{{\left(e_{t}\left(i\right)-\mu_{i}\right)}^{2}}{\sigma_{i}^{2}}$$

In this case, the Mahalanobis distance is effectively reduced to a sum of squared, normalized deviations along each feature dimension. This standardization accounts for the different scales and variances of the individual features, making the anomaly detection more robust. Therefore, we define $s_{t}$ as anomaly score. The anomaly score $s_{t}$ follows a chi-squared distribution with *d* degrees of freedom $s_{t}∼\chi^{2}\left(d\right)$.

The mathematical expectation of the anomaly score is given by $E[s_{t}]=d$. The chi-squared distribution is used to measure the deviation of the residuals from the expected normal behavior. A larger anomaly score indicates a greater deviation from the normal distribution, which can be used to detect abnormal conditions in the time series data.

#### 3.5.3. Threshold Setting

The selection of the threshold τ depends on the $F_{\beta }$ score, which balances the model’s precision (*P*) and recall (*R*). Following standard evaluation metrics for anomaly detection, we define the following parameters:

Precision (*P*) is the proportion of true anomalies among detected anomalies, calculating how many of the samples we predict of a certain type of sample are correctly predicted.

(28) $$P=\frac{TP}{TP+FP}$$

where TP and FP denote true and false positives, respectively.

Recall (*R*) is the proportion of actual anomalies correctly identified, calculating how many samples are correctly predicted for the original actual samples.

(29) $$R=\frac{TP}{TP+FN}$$

where FN as false negatives.

The $F_{\beta }$ score combines *P* and *R* as a weighted harmonic mean:

(30) $$F_{\beta }=\left(1+\beta^{2}\right)\cdot \frac{P\cdot R}{\beta^{2}\cdot P+R}$$

where β adjusts the emphasis on *R* (e.g., β>1 prioritizes recall). The optimal τ maximizes $F_{\beta }$, trading off detection sensitivity (*R*) and reliability (*P*).

#### 3.5.4. Double-Threshold Classification

The optimal threshold τ maximizing $F_{\beta }$ (Equation (30)) provides a single critical value for anomaly declaration. However, to enable early warnings and severity stratification, we generalize this to a double-threshold scheme, which is defined as follows:

(31) $$\rho_{\alpha }=Q_{1-\alpha }\left(s_{\mathrm{train}}\right)$$

where $Q_{1-\alpha }$ denotes the (1−α)-th quantile of the anomaly scores $s_{\mathrm{train}}$ from the training dataset. $\rho_{0.10}$ and $\rho_{0.05}$ represent the thresholds corresponding to the 90th and 95th percentiles of the training scores, respectively.

The anomaly status at time *t* is determined based on the anomaly score. If the anomaly score $s_{t}$ is less than or equal to $\rho_{0.10}$, the state is classified as normal. If the score lies between $\rho_{0.10}$ and $\rho_{0.05}$, the state is marked as suspicious, indicating a possible anomaly. If the score exceeds $\rho_{0.05}$, the state is classified as anomalous, indicating a high likelihood of abnormal behavior.

The double-threshold mechanism provides a nuanced classification, distinguishing between normal, suspicious, and anomalous states based on the severity of the deviation from the normal distribution, supporting the awareness of the device’s situation.

## 4. Blockchain System

The proposed system integrates time series anomaly detection with a blockchain-based mechanism through a synergistic architecture. On the device layer, industrial sensors feed multivariate temporal data to the LEAD module (Section 3), where deep learning models generate anomaly scores while smart contracts automate the execution of response protocols based on anomaly scores, eliminating the need for human intervention in routine fault-handling procedures. The blockchain layer, built upon the FISCO-BCOS [28] consortium chain, establishes an immutable trust anchor by cryptographically binding detection processes with operational outcomes: model hashes and anomaly metadata are permanently recorded on-chain, whereas sensor data remains securely stored off-chain with periodic hash commitments.

### 4.1. Data On-Chain Process

In this system, industrial device sensors periodically collect data and key information is stored on the blockchain to ensure data integrity and traceability. The entire data on-chain process includes data collection, hash computation, identity verification, data storage, and anomaly detection.

A. Data Collection and Pre-processing

As mentioned in Section 3.1, the device data can be represented as a time series D = {x(1),x(2),…,x(t)}, where x(t) represents the device’s situation information at time *t*, such as temperature, pressure, and vibration. The collected data is preprocessed locally to facilitate subsequent hash computation and anomaly detection.

B. Device Data Hash Computation

When a device uploads data *D* to the blockchain, we use the SHA-256 hash function to compute a unique identifier H(D)=SHA-256(D). This hash value ensures data immutability. Even if the raw data is not stored on-chain, its integrity can be verified through hash validation.

C. Device Identity Verification and Data Signing

To prevent unauthorized devices from uploading false data, a digital signature mechanism is used to ensure data authenticity. Suppose the private key of device *i* is $SK_{i}$, then the signature is computed as

(32) $$S_{i}={\mathrm{Sign}}_{SK_{i}}\left(H\left(D\right)\right)$$

When uploading data, the smart contract verifies the signature using the device’s public key $PK_{i}$:

(33) $${\mathrm{Verify}}_{PK_{i}}\left(S_{i},H\left(D\right)\right)$$

If the verification fails, it indicates that the data source is “untrusted”. The contract rejects data storage and triggers the UnauthorizedAttempt event.

#### 4.1.1. Data Storage and Anomaly Detection

**Figure 3:**
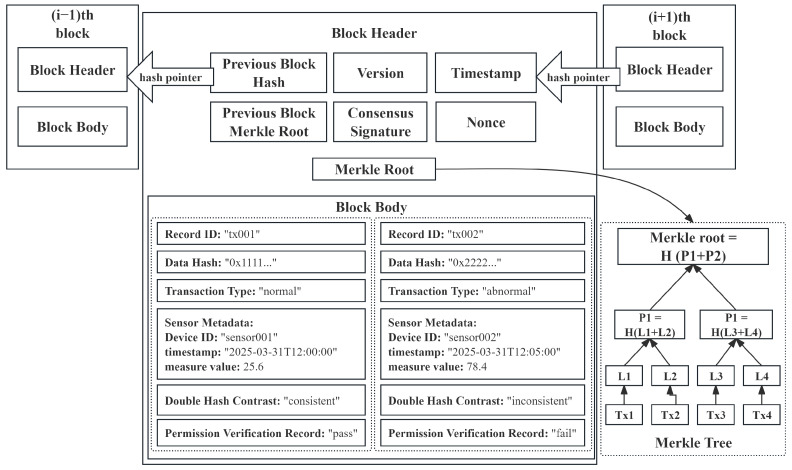
Structure of a block.

Once the identity verification is successful, the hash value H(D) and related metadata (device ID, timestamp *T*) are stored on the blockchain. The structure of the specific data storage is shown in Figure 3. Meanwhile, an LSTM–Attention–LSTM model performs anomaly detection, calculating the anomaly probability $P_{\mathrm{anomaly}}$. If $P_{\mathrm{anomaly}}>\theta$, where θ refers to the classification threshold mentioned in Section 3.5.4, the contract triggers the AnomalyDetected event to notify maintenance personnel to take necessary actions.

#### 4.1.2. Data Integrity Verification and Tampering Detection

To check if data has been tampered with, the device can query the stored hash value $H(D_{\mathrm{blockchain}})$ from the blockchain and compute the local hash value $H(D_{\mathrm{local}})$ for comparison. If the two values do not match, the HashMismatch event is triggered, indicating possible data tampering.

Additionally, a Merkle tree (referring Figure 3) is used to improve data audit efficiency:

(34) $$H_{\mathrm{root}}=H(H\left(D_{1}\right)||H\left(D_{2}\right))$$

**Figure 4:**
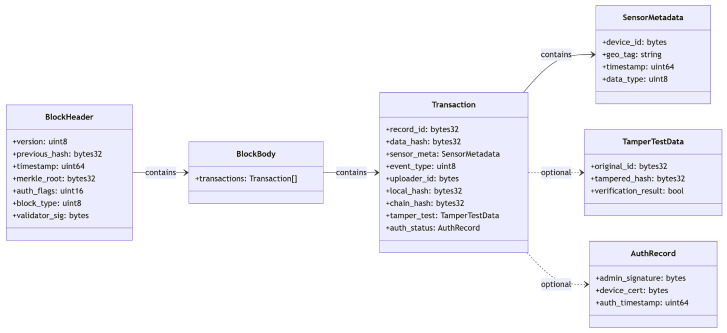
Structure of data storage.

This ensures that, even with a large amount of data stored in a block, data integrity can still be efficiently verified. The above transactions will be recorded in the chain in detail, including data collection, hash computation, identity verification, data storage, and anomaly detection, and the structure of the transaction storage is shown in Figure 4.

### 4.2. Smart Contract Mechanism

FISCO BCOS uses Solidity language to write smart contracts to achieve trusted storage of device status data and exception detection logic. Smart contracts are deployed on the blockchain network. Whenever industrial equipment uploads sensor data, the contract will automatically execute, verify data integrity, and store it on the blockchain. The smart contract also contains exception detection trigger logic, that is, when our LEAD model detects an abnormal device status, the contract will call the notification mechanism, send an alert to the relevant maintenance personnel, and record the event in the blockchain ledger. This automated smart contract execution mode not only reduces human intervention, but also ensures the transparency and tamper resistance of device status data.

We design four smart contracts here, which are responsible for data storage, anomaly detection, identity verification, and data integrity checks, respectively, ensuring a trustworthy record of device status (see Algorithm 2).

**Algorithm 2** Data management contracts
  1:**Data Structure:** 𝒟←(data,hash,τ,addr)  2:
**State Variables:**
    $R:B^{32}\to 𝒟$         ▹ Mapping from record ID to data record    A:A→{0,1}                  ▹ Authorized devices    α∈A                       ▹ Admin address  3:α←msg.sender                      ▹ Contract constructor  4:**function** uploadData(*d*, *h*)  5:    **Require:** onlyAuthorized  6:    $r\leftarrow H(d\|\tau_{\mathrm{block}})$  7:    $R\left[r\right]\leftarrow \left(d,h,\tau_{\mathrm{block}},\mathrm{msg}.\mathrm{sender}\right)$  8:    **Emit** EventUpload(r,msg.sender)  9:
**end function**
10:**function** verifyData(*r*, *d*)11:    **if** R[r].τ=0 **then**12:        **Revert:** “Record invalid”13:    **end if**14:    $h^{\prime }\leftarrow H\left(d\right)$15:    **if** $h^{\prime }\neq R\left[r\right].\mathrm{hash}$ **then**16:        **Emit** EventHashMismatch(r)17:    **end if**18:
**end function**
19:**function** checkAnomaly(*r*, *p*)20:    **if** p>95 **then**21:        **Emit** EventAnomaly(r,p)22:    **end if**23:
**end function**
24:**function** manageDeviceAccess(*a*, mode)25:    **if** mode = admin **then**26:        **if** msg.sender≠α **then**27:           **Revert:** “Access denied”28:        **end if**29:    **else if** mode = authorize **then**30:        **Require:** msg.sender=α31:        A[a]←132:    **else if** mode = revoke **then**33:        **Require:** msg.sender=α34:        A[a]←035:    **else if** mode = check **then**36:        **if** A[a]≠1 **then**37:           **Revert:** “Device unauthorized”38:        **end if**39:    **end if**40:
**end function**



When a device submits data to the blockchain, the contract first verifies whether the device is authorized. If authorized, the data is stored along with its hash, timestamp, and sender address. An anomaly detection mechanism is incorporated to identify potential faults by analyzing anomaly probabilities. If a high anomaly probability is detected, an event is triggered for further investigation.

This dual-layer design not only preserves the temporal sensitivity of LSTM-based detection, but also embeds verifiable audit trails into every decision cycle, transforming statistical anomalies into actionable, tamper-proof events. The contract also supports data auditing, allowing for users to verify whether the stored hash matches the locally computed hash.

## 5. Experiments and Analysis

In order to verify the effect of our system model, our experiment first focuses on the LEAD model to explore its superiority over similar models. Next, we simulate the blockchain system to verify the effectiveness and then analyze its efficiency and security.

### 5.1. Experimental Settings

We use LEAD on the electrocardiogram (ECG) dataset [29], which consists of a univariate ECG signal of two channels that records the electrical activity of the human heart over time. The dataset includes 5000 samples, each consisting of 140 time steps. Among them, only a small proportion represent abnormal heartbeats, introducing a realistic data imbalance similar to that seen in industrial equipment monitoring tasks.

During the data preprocessing stage, time series data serialized in Pickle format is loaded from the specified directory and split into features and labels, where the label corresponds to the last dimension of each data instance. Before training, all data were normalized to the range [0, 1]. We then segmented the signals into fixed-size windows and labeled them according to their ground truth: normal, suspicious, or fault. This preparation allowed us to evaluate our LSTM–Attention anomaly detection model’s ability to identify subtle temporal deviations in time series signals.

We implemented our LEAD model using PyTorch 2.6.0+cu124. The experiments were conducted on a machine with an NVIDIA GPU (CUDA-enabled), and Table 2 shows the hyperparameters used throughout training and evaluation.

During training, the teacher forcing ratio was set to 0.7. We also applied residual connections in the LSTM layers to enhance gradient flow. All models were trained with a fixed random seed (1111) for reproducibility. Anomaly detection was based on reconstruction error, with thresholds determined from training statistics.

To simulate the on-chain mechanism, we use the FISCO-BCOS mentioned above. The operating system is Ubuntu 20.04.6. We import the block structure and smart contract into the blockchain system according to the block design described in Section 4.1 and Section 4.2, and upload simplified sensor data to the chain for the subsequent detection and simulation steps.

### 5.2. Evaluation Index

We use precision, recall, and $F_{\beta }$ to judge the effectiveness of the model. According to Equation (30), when β<1, precision is given more weight; when β>1, recall is given more weight. Here, we choose β=0.1 because, in industrial equipment anomaly detection, data are highly imbalanced (anomalous samples are extremely rare). The proportion of actual anomaly points in sequences labeled as anomalous may not be high, so the recall is expected to be low [30]. Therefore, select the threshold at which the $F_{0.1}$ score is the highest. The values of precision, recall, and $F_{\beta }$ at this threshold are used for evaluation.

### 5.3. Result Analysis

#### 5.3.1. LEAD Prediction

For the training data, the global mean and standard deviation are computed and used to perform z-score standardization, ensuring that each feature dimension has zero mean and unit variance. A Gaussian noise-based data augmentation strategy is employed, in which noise scaled by the standard deviation is added to the original data to generate multiple augmented samples. For test data, the same normalization parameters obtained from the training set are reused, and data augmentation can optionally be applied. We use the LEAD model to predict the future time sequence. Compute the prediction prediction error in order to obtain the anomaly score. The results are then compared with the results of Bi-LSTM [17], EncDec-AD(LSTM) [5] and EncDec-AD(RNN) [10].

**Figure 5:**
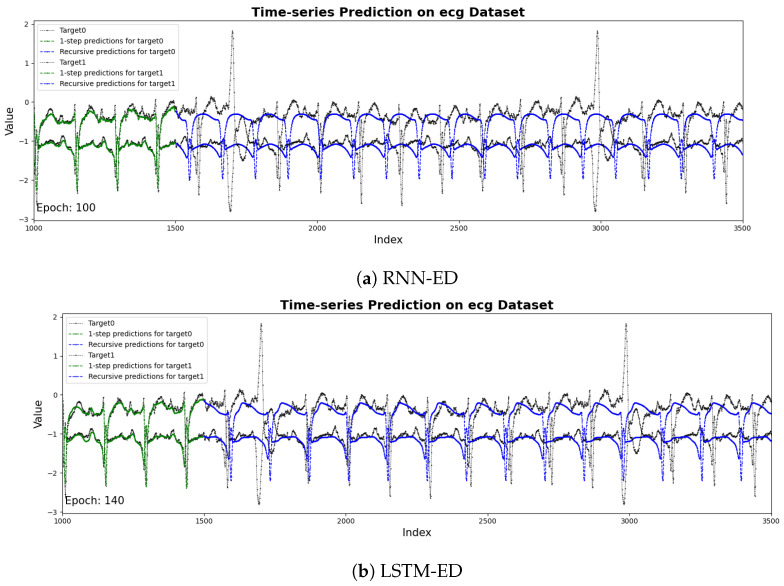

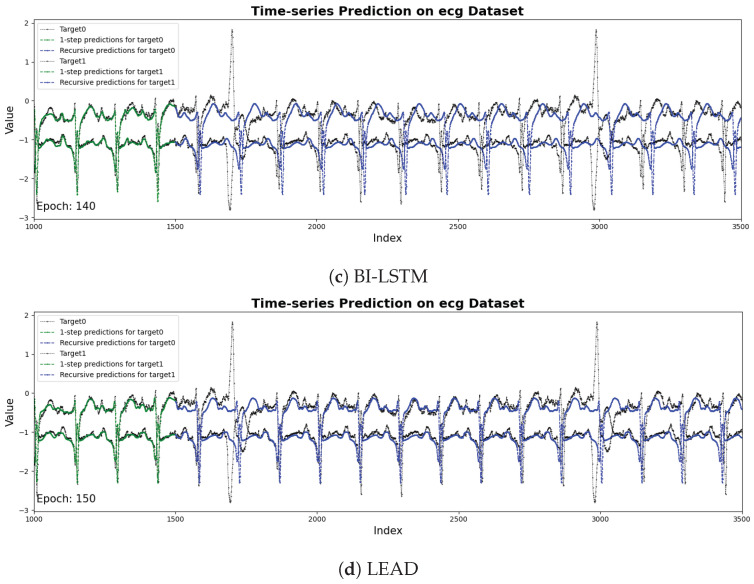
The time-series predictions on ECG using (**a**) RNN, (**b**) LSTM, (**c**) BI-LSTM and (**d**) LEAD models.

Figure 5 shows the results of the prediction sequences by RNN, LSTM, BI-LSTM and LEAD. The two time series represent the two channels in the dataset, respectively. The black line represents the raw sensor data from the target channel, while the blue line denotes the predicted values generated by the model. It is obvious that LEAD performs best in the details of prediction. LEAD has more parameters. The attention mechanism introduces additional weight matrix and computational logic, and the model needs more epochs to fully learn effective time-dependent and contextual information during training. the best can be achieved at the 150th epoch.

#### 5.3.2. Anomaly Detection

**Figure 6:**
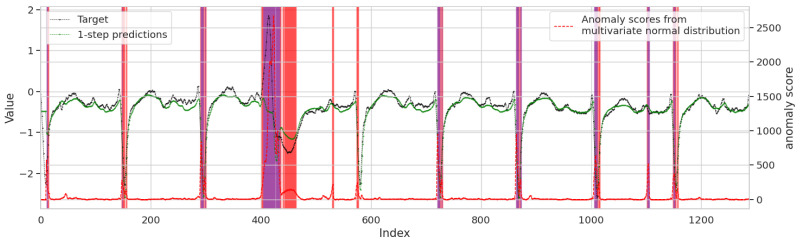
Original sequences with corresponding predictions, as well as the resulting anomaly scores and status markers.

Figure 6 provides a detailed analysis of the LEAD model’s detection results. In the middle panel, the black line and green line represent the target sequence and predicted values, respectively. This sequence is split from one of the two channels in Figure 5 for anomaly detection. The close alignment between these two lines during normal operation demonstrates the model’s predictive accuracy. In the lower panel, the red line illustrates the calculated anomaly scores over time, reflecting the degree of deviation between the actual and predicted values. Regions shaded in red correspond to suspicious windows, where the anomaly score exceeds the predefined suspicion threshold 0.90, indicating potential, but not definitive, anomalies. Regions shaded in purple mark abnormal windows, where the anomaly score surpasses the higher anomaly threshold 0.95. This visualization highlights the LEAD model’s ability to distinguish between slight deviations and true anomalies.

Table 3 shows that all four models—RNN-ED, LSTM-ED, BI-LSTM, and LEAD—achieve 100% precision on both test channels, indicating high reliability in anomaly detection. However, their recall rates vary significantly: 8%, 14%, 13%, and 19%, respectively. Correspondingly, the F_0.1_ scores improve with model complexity, from 0.90 (RNN-ED) to 0.94 (LSTM-ED and BI-LSTM) and 0.96 (LEAD), demonstrating the benefits of architectural enhancements under a precision-focused evaluation. The improvements stem from better temporal modeling: while RNN/LSTM-ED suffer from information compression into fixed-length hidden states, BI-LSTM captures bidirectional dependencies, and the attention mechanism in LEAD further strengthens the model’s ability to focus on early warning patterns.

**Figure 7:**
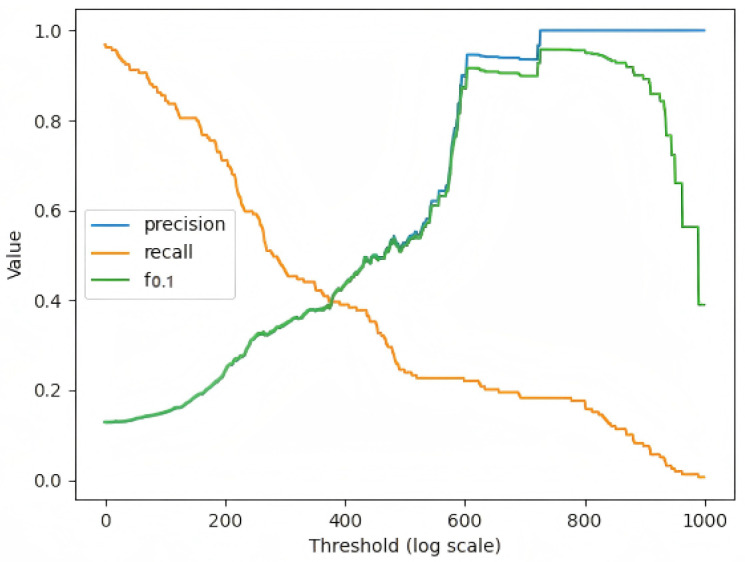
Anomaly detection evaluation $f_{0.1}$.

Figure 7 shows how the $F_{0.1}$ score of the LEAD model changes when different thresholds are taken. We take the maximum value that the recall reaches when the precision reaches 1. In anomaly detection of industrial equipment, there are far fewer abnormal samples than normal samples. There are few abnormal samples in the dataset, and we set threshold to quite high to guarantee the precision. The model tends to predict normally, and the sample with a very high probability will be judged as abnormal, so the precision will be high as 100%, but the recall will be low.

#### 5.3.3. On-Chain Simulation

We deploy the proposed LEAD model within a simulated FISCO BCOS blockchain environment to emulate a real-world IIoT data pipeline. A single-group chain with four or more nodes is configured, and smart contracts are automatically deployed without manual intervention. To evaluate the system’s performance, we design and execute four representative test scenarios: (1) normal data upload, (2) tampered data verification, (3) simulated sensor fault detection, and (4) rejection of unauthorized device access. All testing procedures are fully scripted and automated to reflect realistic IIoT operations.

**Figure 8:**
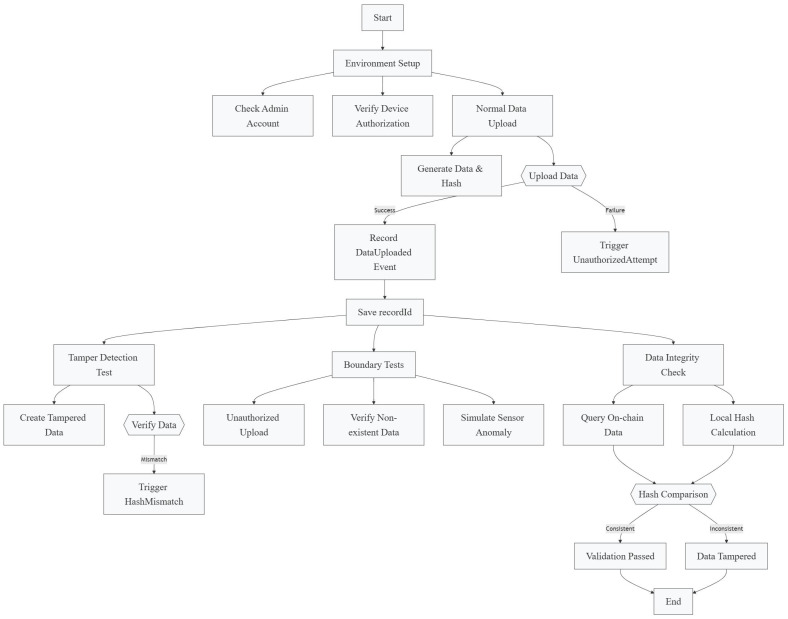
Blockchain simulation process.

Once a transaction is completed, the smart contract emits an event log containing two indexed topics: the first captures the triggered event type, and the second contains the recordID, which serves as a unique key to verify data consistency. Since recordID is defined as an indexed parameter, it is stored within the transaction’s topic log. Figure 8 shows the test process of our transactions. Table 4 gives the results of the three transaction tests that we performed.

Under the consensus, the delay of a single data upload is about 200 ms, which meets the real-time requirements of the industry. When the hash match fails, the HashMismatchevent is triggered (Topic 0: 0x7d7e4bfb...). When a rogue device uploads data, the contract rolls back the transaction (Status 16) through the require statement and triggers the UnauthorizedAttempt event to verify the validity of identity authentication based on digital signature. When the LEAD model detects an anomaly probability $P_{\mathrm{anomaly}}>\theta$, the smart contract triggers the AnomalyDetected event in real-time to notify the maintenance personnel.

**Figure 9:**
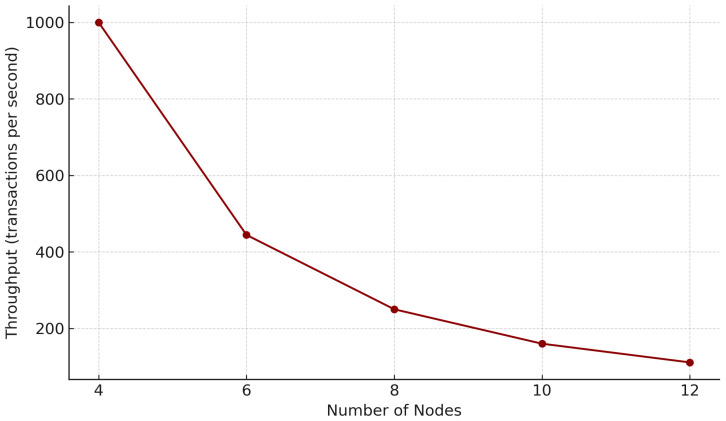
Block throughput vs. nodes.

Figure 9 shows that, as the number of nodes in the FISCO-BCOS network decreases, the network throughput decreases rapidly. This phenomenon stems from the three-stage broadcasting feature of the consensus mechanism. Therefore, this mechanism is more suitable for consortium blockchains with a small number of nodes, and, in practical applications, large-scale nodes can be divided into multiple subgroups (such as five nodes per group). Since the consensus protocol is not the focus of this paper, we will not make any adjustments to it.

## 6. Threats to Validity

There are several potential threats to the validity of our experimental results.

One limitation is the manual selection of anomaly thresholds. Although threshold tuning is common in anomaly detection research, manually set values may introduce subjective bias and affect the consistency of classification results across datasets. In addition, we only evaluated the impact of the multi-head self-attention mechanism, without comparing it to alternative attention variants such as additive or sparse attention. This limits the interpretability of why multi-head attention performs better in our context.

Our model was primarily trained and evaluated on a public ECG benchmark dataset. While this dataset provides clean and labeled sequences for testing, it may not fully reflect the complex operating environments of industrial devices. Furthermore, due to the depth of the proposed model, it requires a relatively large number of training epochs to converge, which may affect reproducibility under constrained hardware or noisy data.

The proposed blockchain integration was tested in a simulated FISCO-BCOS consortium environment with a limited node scale and controlled data upload scenarios. This limits the generalizability of the results to real-world IIoT deployments involving high-throughput, high-frequency sensor networks and unpredictable failure behaviors. The use of ECG data, although structurally similar to other time series, may also reduce generalization to more complex multivariate industrial sensor data.

## 7. Conclusions

This research presents a novel integration of blockchain and deep learning for industrial device fault detection. The LEAD model leverages an attention-enhanced LSTM architecture to capture long-term temporal dependencies in sensor data, while blockchain ensures data immutability through cryptographic hashing and smart contracts. Experimental results validate the model’s superiority in precision and robustness, particularly in scenarios with rare anomalies.

Our study contributes to the literature by bridging two previously separated research streams: deep learning-based fault detection and blockchain-based secure data management. Theoretically, we demonstrate how integrating an LSTM–Attention model with decentralized blockchain infrastructure can improve anomaly detection accuracy while ensuring data integrity and traceability. Practically, the proposed LEAD system can be applied in real-time Industrial IoT environments where equipment failures need to be identified early and securely logged. The blockchain smart contract mechanism ensures unauthorized or tampered data are flagged instantly, which is crucial for safety-critical industries.

In future work, we will optimize the smart contract design to reduce on-chain storage overhead and improve query efficiency. Lightweight inference techniques such as model quantization and knowledge distillation will be explored for edge deployment. Moreover, we will validate the framework on diverse industrial datasets to assess its cross-domain generalizability and robustness under varied conditions.

## Figures and Tables

**Table 1:** Comparison of time series anomaly detection methods based on key metrics.

Evaluation Criteria	Description	Representative Methods and Performance
Temporal Dependency Capture	Ability to model long- and short-term dependencies and nonlinear temporal dynamics	LSTM architectures [9,18] excel in this; traditional methods like SVM [6] are less effective
Anomaly Detection Accuracy & Generalization	Accuracy, recall, and ability to detect novel/unseen anomalies	VAE-LSTM [13], unsupervised autoencoders [11,12] improve generalization but may reduce interpretability; GAN-based models [15] face stability issues
Model Interpretability	Degree to which decision boundaries and anomaly judgments are understandable	Fixed-threshold methods [8] provide high interpretability; deep learning and GAN approaches are less transparent
Real-time Capability & Computational Cost	Suitability for real-time deployment and resource consumption	Basic LSTM and RNN models balance performance and efficiency; ensemble and GAN methods [14,15] have higher computational demands
Automatic Threshold Adaptation	Ability to dynamically adjust anomaly detection thresholds	BiLSTM-Attention with learned thresholds [17] performs well; some methods require manual tuning [16]
Blockchain Integration & Security	Use of blockchain to ensure data integrity and tamper resistance	Blockchain frameworks [23,24] secure data but often introduce latency; optimized approaches [25] reduce overhead but have limited detection scope

**Table 2:** Key experimental hyperparameters.

Parameter	Value	Parameter	Value
Attention heads	4	Input feature dimension	32
Hidden units per layer	32	Number of LSTM layers	2
Sequence length	50 time steps	Batch size	64
Learning rate	0.0002	Dropout rate	0.3
Training epochs	140	Prediction window size	10

**Table 3:** $F_{\beta }$ scores.

Model	Channel	$F_{0.1}$	Precision	Recall
RNN-ED	1	0.90	1	0.08
	2	0.90	1	0.08
LSTM-ED	1	0.94	1	0.13
	2	0.92	1	0.10
BI-LSTM	1	0.94	1	0.14
	2	0.94	1	0.14
LEAD	1	0.96	1	0.19
	2	0.96	1	0.19

**Table 4:** On-chain test results.

Test Scenario	Triggered Event	Event Hash (Topic 0) ^1^	Status (Topic 1)	On-Chain Behavior
Normal Upload	DataUploaded	0x4d238d7e6ecbe9c0d9a0a9a7c4e7c4e2c4c4a5a5a5a5a5a5a5a5a5a5a5a5a5a5a5a	0	Data upload operation is successfully executed
Data Temper	HashMismatch	0x7d7e4bfb4a024e05257653cb1978b93df6c14bb0b54b43100922d03b60df76fc	0	Function logic allows for hash mismatch (only triggers the event without interrupting the transaction)
Unauthorized Device Upload	UnauthorizedAttempt	0x3e8c6d0c4b0c4b0c4b0c4b0c4b0c4b0c4b0c4b0c4b0c4b0c4b0c4b0c4b0c4b0c4b	16 (denied)	Transaction is rolled back by a require statement

^1^ The hash calculation rule is in accordance with Keccak-256.

## Data Availability

The original contributions presented in the study are included in the article; further inquiries can be directed to the corresponding author.
